# Cytokeratin 7-positive/cytokeratin 20-negative cecal adenocarcinoma metastatic to the uterine cervix: a case report

**DOI:** 10.1186/s12957-016-0774-z

**Published:** 2016-01-25

**Authors:** Masafumi Toyoshima, Yuta Momono, Hiromitsu Makino, Takako Kudo, Naomi Oka, Junko Sakurada, Hiroyoshi Suzuki, Hideaki Kodama, Kosuke Yoshinaga

**Affiliations:** 1 Department of Obstetrics and Gynecology, Sendai Medical Center, National Hospital Organization, Sendai, Japan; 2 Department of Pathology, Sendai Medical Center, National Hospital Organization, Sendai, Japan; 3 Department of Surgery, Sendai Medical Center, National Hospital Organization, Sendai, Japan; 4 Present address: Department of Obstetrics and Gynecology, Tohoku University Graduate School of Medicine, 2-8-8, Miyagino, Miyagino-ku, Sendai, Miyagi 983-8520 Japan

**Keywords:** CDX2, Cecal cancer, Cervical metastasis, Cytokeratin 7, Cytokeratin 20, Immunohistochemistry, Tumor marker

## Abstract

**Background:**

The vast majority of uterine cervical malignancies are primary carcinomas, and secondary neoplasms that metastasize to the uterine cervix from a distant organ are uncommon. Although relatively rare, metastases to the uterine cervix from a primary colon cancer have been reported. We report a rare case of metastatic carcinoma originating from a cecal adenocarcinoma with an unusual cytokeratin 7/cytokeratin 20 immunophenotype.

**Case presentation:**

A 74-year-old postmenopausal Japanese woman was referred to our hospital for the evaluation of a uterine tumor. She had a past medical history of cecal cancer and had undergone laparoscopically assisted right hemicolectomy at the age of 69 years. During follow-up, she was found to have elevated levels of the tumor markers carbohydrate antigen 19-9 (179.7 IU/mL) and carcinoembryonic antigen (26.9 μg/L). Positron emission tomography/computed tomography showed a focus of high 18F-fluorodeoxyglucose uptake in her uterus. Examination of a cervical biopsy found a poorly differentiated adenocarcinoma that was immunopositive for cytokeratin (CK)7 and caudal-related homeobox 2 (CDX2) expression and immunonegative for cytokeratin 20 expression. The patient underwent radical hysterectomy and bilateral salpingo-oophorectomy. Histopathological examination found invasive growth of irregular and atypical ductal hyperplasia. Immunohistochemical staining of the tumor specimen revealed the same immunophenotype as the biopsy specimen. The cecal cancer specimen from her previous surgery was also examined and found to have the same immunophenotype. The histopathological diagnosis was cecal adenocarcinoma metastatic to the uterine cervix. The patient is currently receiving adjuvant chemotherapy and to date is without evidence of recurrent disease.

**Conclusions:**

Our report illustrates the importance of immunohistochemistry for the correct diagnosis of the origin of a uterine cervical adenocarcinoma in a patient with a medical history of colorectal cancer. Re-examination of a previous oncological specimen is critical for cases with a uterine lesion that is difficult to identify as primary or metastatic cancer.

## Background

The vast majority of uterine cervical malignancies are primary carcinomas, and other than metastases from the uterine corpus, secondary neoplasms that metastasize to the uterine cervix from other organs are uncommon. Endometrial cancer sometimes spreads to the cervix by direct invasion, with or without stromal involvement. Although relatively rare, distant metastases to the uterine cervix from a primary tumor originating in the ovary, colon, stomach, breast, peritoneum, pancreas, kidney, and renal pelvis have been reported [1–3].

About 15 % of newly diagnosed cases of colorectal adenocarcinoma originate in the cecum [4]. Colorectal cancer, including colorectal cancer arising in the cecum, commonly metastasizes to the liver, lymph nodes, lungs, and peritoneum; but a distant metastasis to the uterine cervix is very rare [2]. It is more likely that an adjacent rectosigmoid cancer will invade the uterine cervix directly or by dissemination [5].

Here, we report a patient with a metastasis to the uterine cervix that originated from a cecal carcinoma removed 5 years previously. This report highlights the usefulness and pitfalls of immunohistochemistry for determining the origin of a cervical lesion in a patient with a history of colorectal cancer.

## Case presentation

**Fig. 1:**
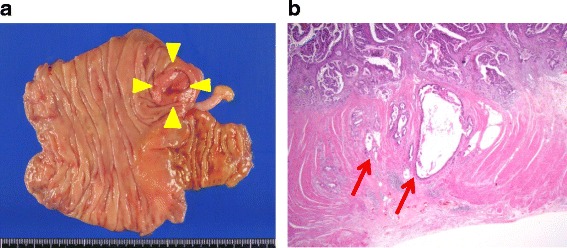
Pathology of the primary cecal carcinoma. **a** Gross appearance of the ulcerous tumor in the cecum (*yellow arrowheads*). Note that a normal appendix is apparent. **b** Hematoxylin-and-eosin-stained section of the primary cecal cancer specimen demonstrates that the adenocarcinoma has slightly invaded the muscle layer to reach the subserosa (*red arrows*) (original magnification ×100)

**Fig. 2:**
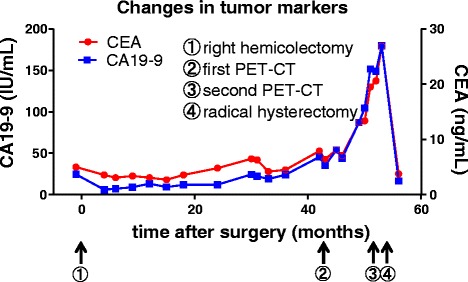
Timeline of clinical events and changes in CEA and CA19-9 levels

**Fig. 3:**
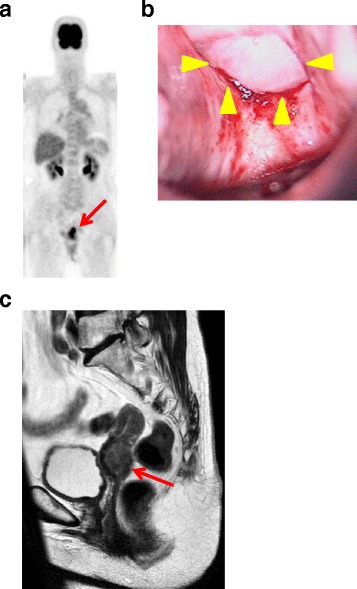
Imaging and physical examinations. **a** Positron emission tomography-computed tomography shows high 18F-fluorodeoxyglucose uptake consistent with a uterine tumor (*red arrow*). **b** Colposcopic view shows a solid white tumor on the posterior fornix (*yellow arrowheads*). **c** Pelvic magnetic resonance image of a T2-enhanced sagittal section showing an irregularly enlarged uterine cervical tumor (*red arrow*)

A 74-year-old postmenopausal Japanese woman was referred to our hospital for the evaluation of a uterine tumor. She was gravida 2, para 2, and otherwise healthy and asymptomatic at the time of presentation. She had a past medical history of mild cerebral infarction in 7 years earlier than her first visit. At the age of 69 years, she was diagnosed with advanced colon cancer at a different hospital and underwent laparoscopically assisted right hemicolectomy with end-to-end anastomosis. Histopathological examination of the neoplasm revealed adenocarcinoma of the cecum, classified as tub1, pSS, ly2, v1, N0, and stage II (Fig. 1a, b). The patient refused adjuvant chemotherapy and was observed as an outpatient. At 42 months of follow-up, she was noted to have the following elevated tumor markers: carbohydrate antigen 19-9 (CA19-9) (45.6 IU/mL) and carcinoembryonic antigen (CEA) (7.9 μg/L). Positron emission tomography-computed tomography (PET-CT) was performed, with no significant findings. Ten months later, the levels of tumor markers were increased (CA19-9, 179.7 IU/mL; CEA, 26.9 ng/mL; Fig. 2). A repeat PET-CT showed a focus of high 18F-fluorodeoxyglucose uptake (standardized uptake value_max_ = 7.83) in her uterus (Fig. 3a), and she was referred to our gynecology department. Colposcopy revealed a tumorous lesion on the posterior fornix (Fig. 3b). Internal and digital examinations were negative for parametrial and rectal involvement. Pelvic magnetic resonance imaging confirmed the presence of a 22-mm mass showing heterogeneous enhancement (Fig. 3c). Laboratory testing revealed normal blood counts and biochemistry. Levels of carbohydrate antigen 125 and squamous cell carcinoma antigen were also within normal ranges. Histopathological examination of a cervical biopsy found a poorly differentiated adenocarcinoma that was positive for cytokeratin (CK)7 and caudal-related homeobox 2 (CDX2) protein expression and negative for CK20 expression. Upper gastrointestinal studies and sigmoidoscopy were negative for tumors.

**Fig. 4:**
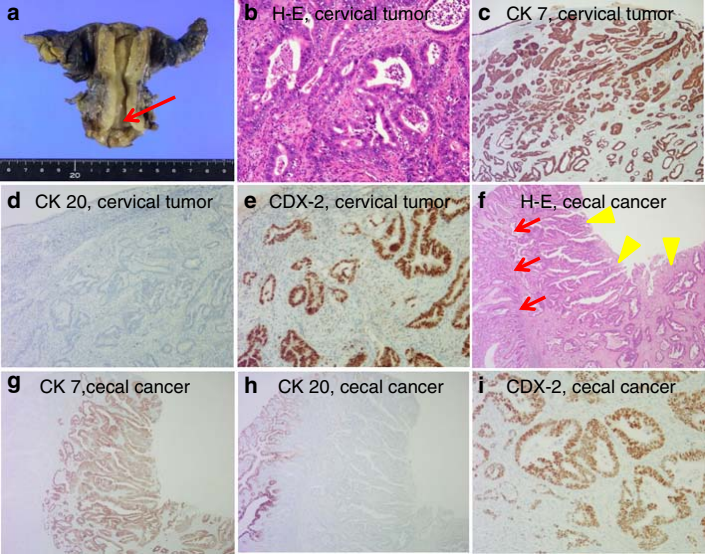
Gross appearance of resected uterus, histopathology, and immunohistochemistry of sections of uterine cervical tumor and cecal tumor specimens. **a** Gross appearance of the resected uterus. Note that the mucosal surface of the endocervical canal is clear (*red arrow*). **b** Hematoxylin and eosin staining of the cervical tumor specimen demonstrates invasive growth of irregular and atypical ductal hyperplasia with moderate differentiation (original magnification ×200). **c** Cervical carcinoma shows diffuse and strongly positive CK7 staining (original magnification ×100). **d** Cervical carcinoma is negative for CK20 staining (original magnification ×100). **e** Cervical carcinoma cells show diffuse and strongly positive CDX2 nuclear staining (original magnification ×200). **f** Hematoxylin-and-eosin-stained section of the cecal adenocarcinoma demonstrates well-differentiated adenocarcinoma (*yellow arrowheads*). Note that normal crypt architecture is seen on the left side of the picture (*red arrows*) (original magnification ×100). **g** Cecal carcinoma shows diffuse and strongly positive CK7 staining (original magnification ×100). **h** Cecal carcinoma is negative for CK20 staining. Note that normal crypt architecture is positive for CK20 staining (original magnification ×100). **i** Cecal carcinoma shows diffuse and strongly positive CDX2 nuclear staining (original magnification ×200)

With a presumptive diagnosis of uterine cervical cancer, whether or not the tumor was primary or metastatic, the patient underwent radical hysterectomy and bilateral salpingo-oophorectomy plus bilateral pelvic lymphadenectomy. Only small peritoneal nodule was found during the procedure. Gross examination of the resected uterus found mild swelling and sclerosis of the posterior cervix, but there was no apparent tumor on the mucosal surface of the endocervical canal (Fig. 4a). The patient’s postoperative course was uneventful, and neurogenic bladder did not develop. Histopathological examination found irregular, invasive growth of atypical ductal hyperplasia (Fig. 4b). The endometrium and cervical grand were atrophic, and there was no endocervical infiltration by the tumor. The excised lymph nodes were negative for metastases, and peritoneal washing fluid was negative for tumor. Immunohistochemical staining of the tumor was positive for CK7 (Fig. 4c), CDX2 (Fig. 4e), and CEA expression and negative for CK20 (Fig. 4d), p16, estrogen receptor, progesterone receptor, vimentin, calretinin, and cluster of differentiation-10 expression. The previous specimens of cecal tumor were also examined after immunohistochemical staining, and the malignant cells manifested the same staining patterns seen for the uterine tumor (Fig. 4g–i). The final histopathological diagnosis was adenocarcinoma of the cecum metastatic to the uterine cervix. The patient is currently receiving adjuvant chemotherapy and to date is without evidence of recurrent disease.

### Discussion

Adenocarcinoma of the cervix accounts for 7 to 22 % of cervical malignancies [6]. Although the vast majority of cervical adenocarcinomas are primary tumors, there are reports and reviews on metastases from distant primaries, including colorectal cancer, to the uterine cervix [1, 2]. Primary cancers of the cecum are anatomically less likely to invade the uterine cervix than primary rectosigmoid cancers. The reasons accounting for the relative rarity of metastasis to the cervix are unclear, but the following histological and anatomical factors may be involved: (a) the uterine cervix is a small structure, with low vascular density in postmenopausal women; (b) the cervix contains a high proportion of fibrous tissue, which may be unsuitable stroma for seeding by metastatic cells; and (c) all pelvic lymphatic vessels drain away from the cervix. The evidence from our rare case was consistent with a metastasis to the uterine cervix that originated from a distant tumor. The metastatic lesion was subsequently treated by surgery, which obtained clear resection margins.

The best treatment for a patient with a large cervical metastasis from another organ depends on the patient’s physical condition, characteristics of the tumor, and medical findings. Generally, for a patient with metastatic cervical tumor such as ours, the decision to perform surgical resection should be made with careful deliberation, because there is no evidence showing apparent clinical benefit for such cases. In addition, radical hysterectomy with lymph node dissection is a highly invasive surgery. Despite the possibility that the patient had metastatic cecal cancer, we decided to perform surgery taking into consideration that the patient’s systemic evaluation, including PET-CT, provided evidence that she had a solitary uterine lesion. In addition, the latest guidelines for colon cancer patients from the National Comprehensive Cancer Network (NCCN guideline, Colon Cancer, version 3.2015) recommend resection as a primary treatment for patients with resectable metastatic disease. Adjuvant chemotherapy should be considered for patients, including ours, who have not received previous chemotherapy.

CEA and CA19-9 are established tumor markers used for the preoperative diagnosis and detection of recurrent disease in patients with colorectal cancer. Both tumor markers are useful, especially for the postoperative follow-up of patients who are treated with chemotherapy, because, in most cases, increasing serum levels of both markers indicate recurrence or metastatic lesions. Kuusela et al. reported that elevated CA19-9 levels showed very high specificity (97 %) and positive predictive value (98 %) for patients with colorectal carcinoma, but its sensitivity was only around 45 %, even for patients with recurrent colorectal cancer [7]. However, the combination of CA19-9 with CEA has a sensitivity of 82.5 % (33/40) for patients with relapsed colorectal cancer [7]. The diagnostic sensitivities of CEA and CA19-9 for primary cervical adenocarcinoma were reported to be 38.5 and 41.7 %, respectively [8]. Although the levels of CA19-9 and CEA were within normal ranges at the time our patient underwent right hemicolectomy (Fig. 2(①)), both tumor markers became gradually elevated (Fig. 2(②)) and rapidly increased just before the second PET-CT revealed tumor in her uterine cervix (Fig. 2(③)). Furthermore, both CA19-9 and CEA rapidly returned to levels within normal limits after the patient underwent radical hysterectomy (Fig. 2(④)). These changes indicate that CA19-9 and CEA were useful markers in the clinical course of our case.

The correct diagnosis of a lesion in a patient with a past history of cancer, which differentiates between primary and metastatic disease, is very important for determining the stage of disease, appropriate treatment, and prognosis. However, performing a histopathological diagnosis that discriminates between a colorectal adenocarcinoma metastatic to the cervix and a primary cervical adenocarcinoma with colonic differentiation is difficult [9]. Because the morphological features of primary and metastatic tumors can overlap, a differential diagnosis based on immunohistochemical staining that can distinguish between these two types of lesions is essential. Determining the expression of CK7 and CK20 is one of the most useful diagnostic options. CK7 is expressed by many ductal and glandular epithelial cells (mainly gallbladder, hepatic ducts, and pancreatic ducts), by tissues in the female genital tract (ovary, endometrium, fallopian tube, and cervix), and by tissues in the breast, lung, and urinary tract. On the other hand, CK20 expression is restricted to a few organ systems, and CK20 positivity has been found in urothelial tumors; Merkel cell tumors; adenocarcinomas of the stomach, biliary duct, and pancreas; and, most importantly, almost all colon carcinomas [10]. Many lesions of colorectal origin have a CK7-negative/CK20-positive immunohistochemical profile, which is reversed in tumors of gynecological origin, including the uterine cervix. Bayrak et al. reported that a CK7-positive/CK20-negative expression pattern was observed in only 1.7 % (2 of 118) of colorectal carcinomas [10]. In addition, McCluggage et al. reported that all cervical adenocarcinoma and adenocarcinoma in situ samples (38/38) were diffusely positive for CK7 [11]. In our case, the immunohistochemistry of our patient’s cervical biopsy and surgical specimen showed a CK7-positive/CK20-negative expression pattern (Fig. 4c, d). Therefore, we initially speculated that the possibility of metastatic carcinoma from the cecum was low, although CDX2 was positive in both samples (Fig. 4e).

CDX2 is a homeobox transcription factor that regulates the differentiation of intestinal epithelial cells [12]. Previous studies have shown that CDX2 protein is expressed in the nuclei of normal and malignant intestinal epithelial cells and is highly sensitive for the identification of colorectal adenocarcinoma [13]. However, CDX2 expression may also be positive in adenocarcinomas of the upper gastrointestinal tract and other lesions, including those of the uterine cervix. CDX2-positive nuclear staining has been reported to occur in 71.8 to 99 % of colorectal cancers [10, 14, 15] and in 5.8 to 30 % of cervical adenocarcinomas [13, 14]. We think that an immunohistochemical panel of CK7, CK20, and CDX2 can be helpful in the differential diagnosis of the possible origin of the metastasis and primary cervical cancer.

## Conclusions

To the best of our knowledge, we presented the first case in the English literature of a patient with a metastasis to the uterine cervix that originated from a cecal adenocarcinoma with an unusual CK7-positive/CK20-negative immunophenotype. Our report illustrates the importance of immunohistochemistry for the correct diagnosis of the origin of a uterine cervical adenocarcinoma in a patient with a medical history of colorectal cancer. Re-examination of a previous oncological specimen is critical for cases with a uterine lesion that is difficult to identify as primary or metastatic cancer. Also, we emphasize the importance of monitoring the tumor markers: CA19-9 and CEA, to assess recurrence of colon cancer.

### Consent

Written informed consent was obtained from the patient for publication of this case report and accompanying images. A copy of the written consent is available for review by the Editor-in-Chief of this journal.

## Abbreviations

CA19-9: carbohydrate antigen 19-9
CDX2: caudal-related homeobox 2
CEA: carcinoembryonic antigen
CK: cytokeratin
PET-CT: positron emission tomography-computed tomography

## References

[1] Lemoine NR, Hall PA (1986). Epithelial tumors metastatic to the uterine cervix. A study of 33 cases and review of the literature. Cancer.

[2] Nakagami K, Takahashi T, Sugitani K, Sasaki T, Ohwada S, Morishita Y (1999). Uterine cervix metastasis from rectal carcinoma: a case report and a review of the literature. Jpn J Clin Oncol.

[3] McCluggage WG, Hurrell DP, Kennedy K (2010). Metastatic carcinomas in the cervix mimicking primary cervical adenocarcinoma and adenocarcinoma in situ: report of a series of cases. Am J Surg Pathol.

[4] Vukasin AP, Ballantyne GH, Flannery JT, Lerner E, Modlin IM (1990). Increasing incidence of cecal and sigmoid carcinoma. Data from the Connecticut Tumor Registry. Cancer.

[5] Chereau E, Ballester M, Gonin J, Lesieur B, Darai E (2011). Cervical metastasis from colorectal cancer. World J Oncol.

[6] Hebblethwaite N, Boyd K, Peel KR, Williamson PR, Melling PP, Smith AD (1997). Adenocarcinoma of the cervix: a regional retrospective study. Eur J Gynaecol Oncol.

[7] Kuusela P, Jalanko H, Roberts P, Sipponen P, Mecklin JP, Pitkänen R (1984). Comparison of CA 19-9 and carcinoembryonic antigen (CEA) levels in the serum of patients with colorectal diseases. Br J Cancer.

[8] Borras G, Molina R, Xercavins J, Ballesta A, Iglesias J (1995). Tumor antigens CA 19.9, CA 125, and CEA in carcinoma of the uterine cervix. Gynecol Oncol.

[9] Raspollini MR, Baroni G, Taddei A, Taddei GL (2003). Primary cervical adenocarcinoma with intestinal differentiation and colonic carcinoma metastatic to cervix: an investigation using Cdx-2 and a limited immunohistochemical panel. Arch Pathol Lab Med.

[10] Bayrak R, Haltas H, Yenidunya S (2012). The value of CDX2 and cytokeratins 7 and 20 expression in differentiating colorectal adenocarcinomas from extraintestinal gastrointestinal adenocarcinomas: cytokeratin 7-/20+ phenotype is more specific than CDX2 antibody. Diagn Pathol.

[11] McCluggage WG, Shah R, Connolly LE, McBride HA (2008). Intestinal-type cervical adenocarcinoma in situ and adenocarcinoma exhibit a partial enteric immunophenotype with consistent expression of CDX2. Int J Gynecol Pathol.

[12] James R, Kazenwadel J (1991). Homeobox gene expression in the intestinal epithelium of adult mice. J Biol Chem.

[13] Sullivan LM, Smolkin ME, Frierson HF, Galgano MT (2008). Comprehensive evaluation of CDX2 in invasive cervical adenocarcinomas: immunopositivity in the absence of overt colorectal morphology. Am J Surg Pathol.

[14] De Lott LB, Morrison C, Suster S, Cohn DE, Frankel WL (2005). CDX2 is a useful marker of intestinal-type differentiation: a tissue microarray-based study of 629 tumors from various sites. Arch Pathol Lab Med.

[15] Werling RW, Yaziji H, Bacchi CE, Gown AM (2003). CDX2, a highly sensitive and specific marker of adenocarcinomas of intestinal origin: an immunohistochemical survey of 476 primary and metastatic carcinomas. Am J Surg Pathol.

